# Shared genetic architecture of smoking dependence and Crohn's disease: A cross-trait analysis of GWAS summary statistics

**DOI:** 10.18332/tid/231217

**Published:** 2026-09-11

**Authors:** Aocheng Ji, Wenbin Xu, Hao Xiong, Huan Zhong, Chunyan Zeng

**Affiliations:** 1 Department of Gastroenterology, Jiangxi Province Hospital of Integrated Chinese and Western Medicine, Nanchang, China; 2 Eye Hospital of China Academy of Chinese Medical Sciences, Beijing, China; 3 Department of Biochemistry and Molecular Biology, University of British Columbia, Vancouver, Canada

**Keywords:** Crohn's disease, smoking dependence, PLACO, gsMap

## Abstract

**INTRODUCTION:**

Smoking dependence (SD) and Crohn's disease (CD) are epidemiologically associated, but whether this relationship reflects shared genetic susceptibility remains unclear.

**METHODS:**

We conducted a cross-trait genetic analysis of SD and CD using publicly available genome-wide association study (GWAS) summary statistics from European-ancestry populations. Genome-wide genetic correlation was estimated using linkage disequilibrium score regression (LDSC) and high-definition likelihood (HDL). Pleiotropic variants were identified using PLACO and mapped to genomic loci using FUMA. Regional signal sharing was assessed by Bayesian colocalization. Functional analyses included stratified LDSC, Multi-marker Analysis of GenoMic Annotation (MAGMA), GTEx tissue analysis, and Metascape. Expression-linked candidate genes were prioritized using expression quantitative trait locus (eQTL)-based summary-data-based Mendelian randomization (SMR) with heterogeneity in dependent instruments (HEIDI) testing. Genetically informed spatial mapping of cells for complex traits (gsMap) was used for spatial mapping.

**RESULTS:**

SD and CD showed positive genetic correlation by LDSC (rg=0.2090, p=0.0008) and HDL (rg=0.3817, p=0.00106). PLACO identified 81 genome-wide significant pleiotropic SNPs, which were mapped by FUMA to three loci at 1p31.3, 5p13.1, and 12q12, represented by rs11209031, rs1395152, and rs17467116, respectively. MAGMA identified 22 FDR-significant genes, four of which remained Bonferroni significant: *LRRK2, TNFRSF6B, ZGPAT,* and *RP4-583P15.15*. Cross-trait tissue analysis showed significant enrichment of the shared genetic signal in whole blood and small intestine, while gene-set analysis highlighted inflammatory response (p_bon_=1.86×10^-5^) and T-helper 17 cell differentiation (p_bon_=7.37×10^-4^). SMR/HEIDI analysis further prioritized *RPS6KB1* as a shared expression-linked candidate. Spatial mapping revealed a prominent signal in the embryonic gastrointestinal tract and gene-specific regional patterns involving *LRRK2* and *SLC2A13* in the adult mouse brain.

**CONCLUSIONS:**

SD and CD showed measurable shared genetic susceptibility, with convergent evidence from pleiotropic loci, immune-inflammatory pathway enrichment, tissue-level associations, and spatial transcriptomic mapping.

## INTRODUCTION

Crohn’s disease (CD), which exhibits a substantial genetic contribution, is a chronic immune-mediated gastrointestinal disorder^1^. Beyond hallmark symptoms such as abdominal pain, diarrhea, and fever, CD is notable for its relapsing course and lack of curative treatment. These clinical characteristics may contribute to a markedly elevated risk of psychiatric comorbidities, with up to 18.3% of patients developing anxiety disorders^2^. Some evidence suggests that active CD is associated with a greater burden of psychiatric and substance-use comorbidities. For instance, pharmacological treatment for substance-use disorders, including tobacco use, was more frequent among patients with active than inactive CD (19.8% vs 4.3%)^2^.

Smoking dependence (SD) is a mental and behavioral disorder characterized by strong psychological and physiological reliance on nicotine^3^. The transition from initial smoking behavior to full-blown dependence is driven not only by nicotine-induced upregulation of nicotinic acetylcholine receptors in the central nervous system, but also by genetic predisposition^4^. In addition to well-known risk genes such as the *CHRNA4*, *CHRNB2*, and *CHRNA5/A3/B4* gene clusters, recent studies have revealed a close association between the *CHRNB3-CHRNA6* gene cluster and nicotine dependence, with supporting evidence from gene-knockout mouse models^5^. Moreover, a significant correlation between single-nucleotide polymorphisms (SNPs) in this region and the number of quit attempts has been identified, providing a genetic explanation for individual differences in tobacco dependence and treatment response^6,7^.

A recent genome-wide association study (GWAS) based on the Fagerström test for nicotine dependence (FTND) estimated the SNP-based heritability of SD to be approximately 8.6%^6^. Notably, novel SNPs near *MAGI2/GNAI1* (7q21), *TENM2* (5q34), and *ARHGAP25* (2p13) were identified as potential loci that specifically influence nicotine dependence rather than smoking quantity^6^.

**Figure 1:**
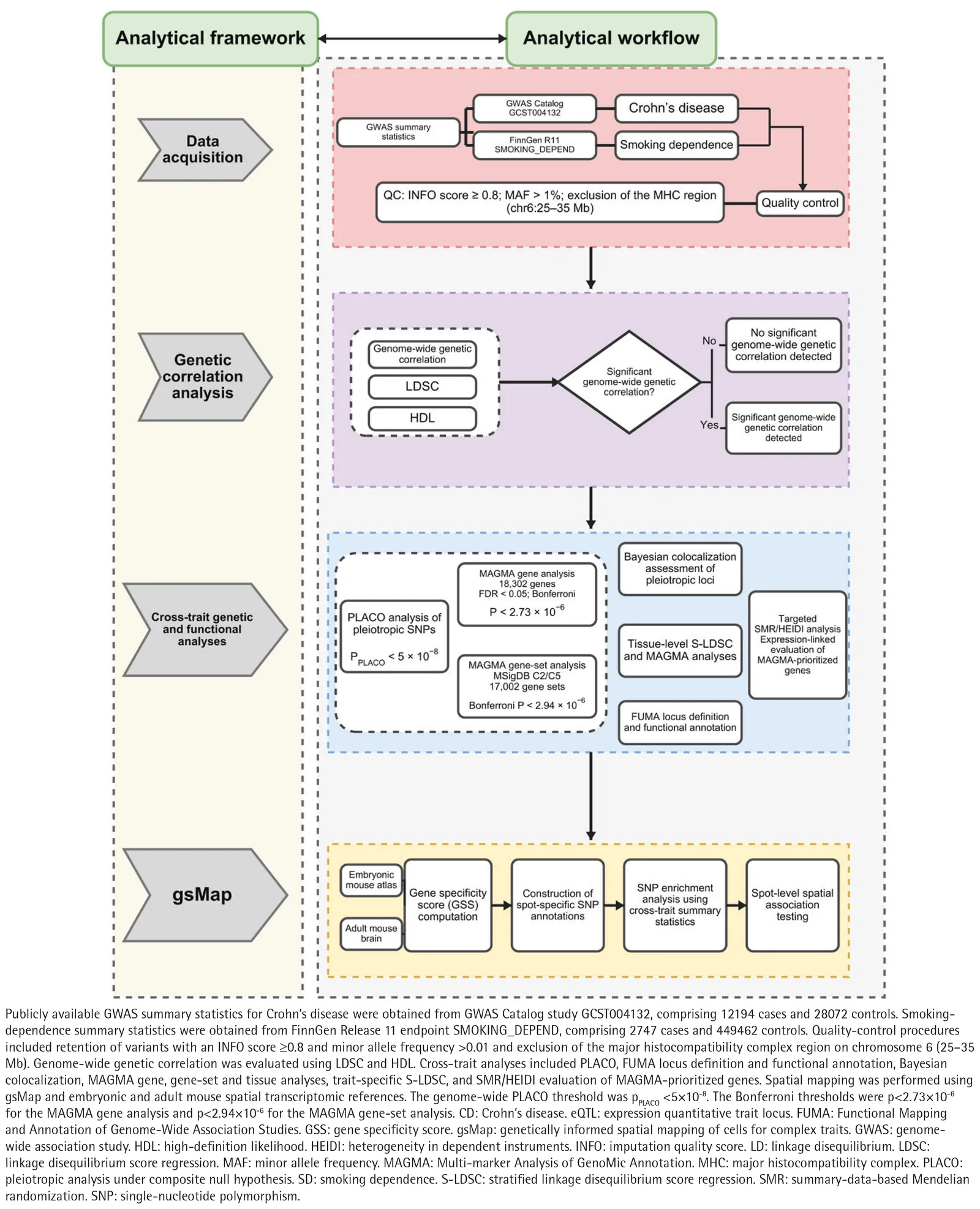
Analytical workflow for the cross-trait GWAS summary-statistics analysis of Crohn’s disease and smoking dependence using European-ancestry datasets (CD: GWAS Catalog GCST004132, 2017, N=40266; SD: FinnGen R11, 2024, N=452209)

Although smoking cessation is widely recognized as an important component of the clinical management of CD, the potential shared genetic basis of smoking dependence and CD remains insufficiently characterized. To bridge this knowledge gap, we systematically characterized the shared genetic architecture of SD and CD. We assessed their genome-wide genetic overlap, characterized shared susceptibility loci and regional signal sharing, delineated the functional and spatial transcriptomic landscape of these signals, and prioritized expression-linked candidate genes (Figure 1).

## METHODS

### Study design

We employed an integrative cross-trait genetic analytical framework to investigate the shared genetic basis between CD and SD. Initially, we evaluated their genome-wide genetic correlation using linkage disequilibrium score regression (LDSC) and high-definition likelihood (HDL). Subsequently, the Pleiotropic Analysis under Composite Null Hypothesis (PLACO) method was used to identify pleiotropic loci shared between CD and SD. Furthermore, we used Functional Mapping and Annotation of Genome-Wide Association Studies (FUMA) to map and functionally annotate the PLACO-identified loci and conducted Bayesian colocalization analysis. Additionally, Multi-marker Analysis of GenoMic Annotation (MAGMA) was used to further characterize the gene-level associations and biological context of the shared genetic signals. Notably, we applied genetically informed spatial mapping of cells for complex traits (gsMap) to examine the anatomical distribution of the prioritized genetic signals. Finally, expression-linked candidate genes were prioritized by integrating GWAS and eQTL evidence.

### Data sources

All data used in this study were obtained from publicly available resources. GWAS summary statistics for CD were obtained from the GWAS Catalog (GCST004132; 12194 cases and 28072 controls; n=40266)^8^. Summary statistics for SD were obtained from FinnGen release R11 (SMOKING_DEPEND; 2747 cases and 449462 controls; n=452209)^9^. All participants were of European ancestry.

### Data quality control

We implemented several quality-control filters to improve the reliability of GWAS results. First, SNPs located in the major histocompatibility complex (MHC) region on chromosome 6 (25–35 Mb) were excluded. Second, we restricted the analyses to SNPs with a minor allele frequency (MAF) >0.01 to increase statistical power and reduce false positives. Finally, we applied an imputation info score filter and removed SNPs with INFO <0.8 to ensure that the association results were based on well-imputed genotypes^10^.

### Genome-wide genetic correlation

To assess the shared genetic architecture between SD and CD, we employed the LDSC method^11^. LDSC uses GWAS summary statistics and LD information to estimate SNP-based heritability and genome-wide genetic correlation between traits^12^. The LD scores used in the LDSC analysis were based on the 1000 Genomes European phase 3 project^13^. Standard errors (SE) were estimated using the block jackknife procedure implemented in LDSC^11^. Additionally, as a sensitivity analysis, we estimated the genetic correlation using LDSC with an unconstrained cross-trait intercept, allowing the intercept to account for residual inflation due to confounding such as population stratification and cryptic relatedness^12^. To further validate the LDSC results, we applied the high-definition likelihood (HDL) method^14^ (V1.4.1), a likelihood-based analysis tool assuming a multivariate normal distribution. HDL allows for more efficient utilization of GWAS summary statistics and provides more precise estimates of genetic correlations. Specifically, HDL reduces the variance of genetic correlation estimates by approximately 60%, substantially enhancing the precision and robustness of the findings^14^.

### SNP-level prioritization of shared susceptibility

To identify specific genetic variants underlying the significant genetic correlations between SD and CD, we performed a pleiotropy analysis at the SNP level using the PLACO R package (v0.1.1; R v4.3.3)^15^. PLACO is a statistical method designed to detect genetic variants that influence multiple traits, i.e. pleiotropy. This method tests a composite null hypothesis that a genetic variant is either not associated with any traits or associated with only one trait, with the alternative hypothesis being that the variant is associated with both traits. The core test statistic is based on the product of the Z-scores for each SNP from the two GWAS summary datasets. PLACO asymptotically approximates the null distribution of this statistic, and can account for correlation between GWAS summary statistics, including that arising from sample overlap. In practice, we first harmonized effect alleles across the two GWAS datasets, and SNPs reaching genome-wide significance (p_PLACO_ <5×10^-8^) were considered pleiotropic variants. We subsequently used FUMA to define genomic risk loci and annotate the potential functional consequences of the pleiotropic SNPs^16^.

Finally, to determine whether the SD and CD associations observed at shared loci were driven by the same causal variants, we performed Bayesian colocalization analysis^17^ (v5.1.0; R v4.3.3). For each pleiotropic locus, we performed colocalization within the locus boundaries defined in the FUMA GenomicRiskLoci table and estimated posterior probabilities for five mutually exclusive hypotheses (H0–H4): H0, neither trait associated; H1, trait 1 only; H2, trait 2 only; H3, both traits associated with distinct causal variants; and H4, both traits associated with a shared causal variant. We interpreted PP4 ≥0.80 as strong support for a shared causal variant and 0.50 ≤ PP4 <0.80 as moderate support. PP4/PP3 was used as a complementary measure of H4 versus H3 support, with ratios >2.5 considered suggestive only when absolute PP4 support was insufficient.

### Organ-level analysis

To further explore the distribution patterns of the shared genetic basis between SD and CD across different physiological systems, we examined the enrichment of SNP-based the heritability in specific tissues and cell types. We used the S-LDSC method^18^, leveraging GTEx tissue-specific annotations^19^, to assess tissue-specific heritability enrichment. This approach allowed us to systematically assess the statistical significance of the enrichment of genetic heritability for SD and CD in each tissue or cell type.

### Gene-level exploratory analysis

To further characterize the biological relevance of genetic loci shared between SD and CD, we extended the analyses from the SNP level to the gene level. First, using FUMA and physical proximity, we mapped lead SNPs at each locus, together with their linked SNPs, to nearby genes. In parallel, we applied MAGMA^20^ to perform gene-based association analysis, aggregating multiple variants within each gene while accounting for linkage disequilibrium between markers, in order to capture cumulative polygenic effects. Gene-level significance was evaluated using both FDR and Bonferroni correction across 18302 genes. Genes with FDR <0.05 were considered FDR-significant, whereas p<2.73×10^-6^ defined the more stringent Bonferroni threshold (p<0.05/18302=2.73×10^-6^). The 22 FDR-significant genes were carried forward to targeted SMR/HEIDI analyses. We next systematically evaluated the functional characteristics and tissue distribution of these mapped genes. Using MAGMA gene-set enrichment analysis, we tested whether genes within predefined gene sets from MSigDB showed stronger gene-level associations^20,21^, including curated gene sets (C2.all) and Gene Ontology categories for biological process, cellular component, and molecular function (C5.BP, C5.CC, C5.MF)^21^. To control for multiple comparisons, Bonferroni correction was applied across all tested gene sets (p<0.05/17002=2.94×10^-6^). In parallel, we used Metascape^22^ to integrate and visualize pathway enrichment results based on MSigDB, facilitating biological interpretation of the mapped genes. Finally, using expression data from 54 human tissues in GTEx^19^, we performed MAGMA gene-property analysis to assess tissue associations with the cross-trait gene-level genetic signal.

### Expression-linked prioritization of candidate genes using SMR/HEIDI

We applied summary-data-based Mendelian randomization (SMR) to integrate GWAS signals with expression quantitative trait loci (eQTL) data and to evaluate expression-linked support for MAGMA-prioritized genes^23^. For each probe, the top cis-eQTL was used as the instrumental variant. Heterogeneity in dependent instruments (HEIDI) test was applied to assess heterogeneity attributable to linkage, with p_HEIDI_ *>*0.05 interpreted as no statistically significant heterogeneity^23^. By combining SMR with the HEIDI test, we evaluated expression-linked evidence separately in SD and CD across prespecified eQTL panels. SMR p-values were adjusted using the Benjamini–Hochberg FDR procedure across 114 successful tests pooled across SD and CD. Genes supported in both traits after multiple-testing correction and without evidence of HEIDI heterogeneity were considered as shared expression-linked candidates (p_SMR_<0.05, FDR-adjusted <0.05, p_HEIDI_ >0.05).

### Spatial transcriptomic mapping of pleiotropic loci using gsMap

To further characterize the spatial context of pleiotropic genetic signals identified by PLACO, we performed gsMap^24^. Specifically, we projected cross-trait pleiotropic signals onto two high-resolution spatial transcriptomic reference datasets: the E16.5 stage, embryo section 1, sample 1 from the mouse organogenesis spatiotemporal transcriptomic atlas^25^, which provides whole-embryo coverage during mid-gestation, and the adult mouse brain coronal section^26^, which captures fine-grained spatial expression patterns in the adult mouse brain. This dual reference design enabled us to interrogate both developmental and adult tissue contexts.

Within gsMap, gene specificity scores (GSSs) were calculated for individual spatial spots by integrating gene-expression profiles with spatial information. The GSSs were subsequently assigned to nearby SNPs to construct spot-specific SNP annotations. These annotations were integrated with the PLACO-derived cross-trait genetic statistics using S-LDSC to estimate spot-level associations. Regional associations were further summarized by aggregating spot-level p-values using the Cauchy combination test. For descriptive gene-level visualization in the adult mouse brain reference, we selected FUMA-mapped genes spanning the three pleiotropic loci: *MIER1* at 1p31.3, *DAB2* at 5p13.1, and *LRRK2* and *SLC2A13* at 12q12. Among the FUMA-mapped genes at 5p13.1, *DAB2* showed the highest adult-brain gsMap diagnostic PCC. These gene-level maps were descriptive and were not used as an additional gene-prioritization test.

## RESULTS

### Genetic correlation between smoking dependence and Crohn’s disease

We first assessed the genetic correlation between SD and CD (Supplementary file Tables S1 and S2). Using LDSC, we observed a significant positive genetic correlation between SD and CD (rg=0.2090, SE=0.0625, p=0.0008); the estimate was unchanged in the unconstrained-intercept sensitivity analysis, with a cross-trait intercept of 0.0012 (SE=0.0056). Using the HDL method, we obtained consistent results (rg=0.3817, SE=0.1166, p=0.00106) (Table 1).

**Table 1:** Genome-wide genetic correlation between Crohn’s disease and smoking dependence in a cross-trait analysis of publicly available European-ancestry GWAS summary statistics (CD: GWAS Catalog GCST004132, 2017, N=40266; SD: FinnGen R11, 2024, N=452209)

*Trait pairs*	*LDSC*		*HDL*	
	rg (SE)	p	rg (SE)	p
SD and CD	0.2090 (0.0625)	0.0008	0.3817 (0.1166)	0.00106

Genetic correlations (rg) with corresponding standard errors and p-values were estimated using linkage disequilibrium score regression and high-definition likelihood. CD: Crohn’s disease. SD: smoking dependence. GWAS: genome-wide association study. LDSC: linkage disequilibrium score regression. HDL: high-definition likelihood. rg: genetic correlation. SE: standard error. N: sample size.

### Organ-level associations

**Figure 2:**
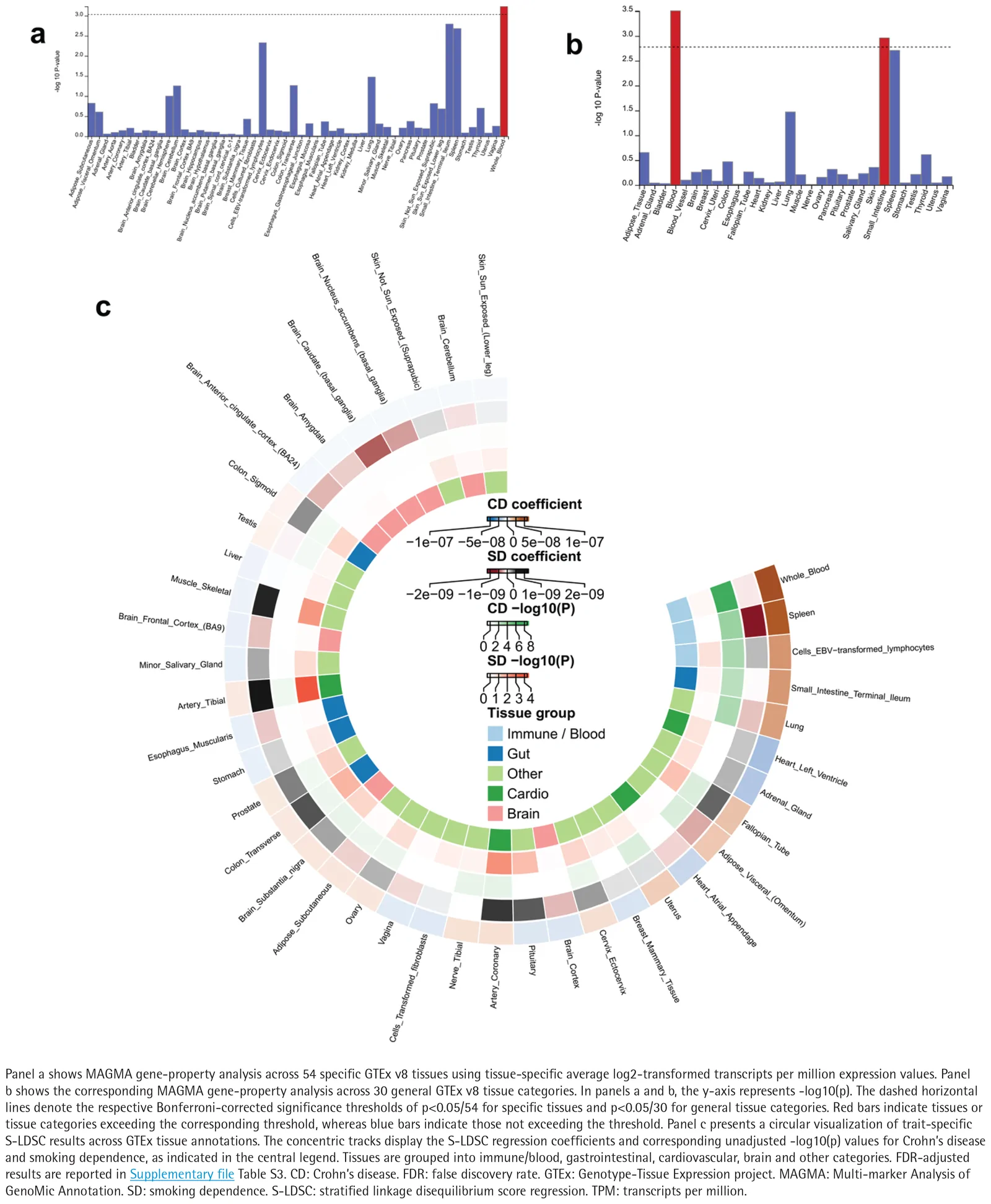
Tissue-level characterization of genetic signals for Crohn’s disease and smoking dependence in a cross-trait analysis of European-ancestry GWAS summary statistics (CD: GWAS Catalog GCST004132, 2017, N=40266; SD: FinnGen R11, 2024, N=452209)

To examine how genetic signals for CD and SD are distributed across tissues and organs, we used S-LDSC to test tissue-specific enrichment of SNP effects. Using GTEx tissue-specific annotations, we fitted GWAS summary statistics for each trait in a model that included the baseline annotations and all gene sets, and evaluated enrichment using the Z scores and p-values of the regression coefficients. SD signals were significantly enriched in the tibial artery (FDR=0.017). CD signals showed significant enrichment in whole blood (FDR=2.14×10^-5^), spleen, EBV-transformed lymphocytes, lung, and terminal ileum, and these enrichments remained significant after FDR correction (FDR<0.05) (Figure 2c; and Supplementary file Table S3).

### MAGMA-based gene-level enrichment analysis

We next carried out gene-level enrichment analysis for CD and SD using the MAGMA module implemented in the FUMA platform. In total, 22 genes passed the significance threshold after FDR correction (FDR <0.05), of which four remained significant after Bonferroni correction: *LRRK2*, *TNFRSF6B*, *ZGPAT*, and *RP4-583P15.15*. MAGMA gene-set analysis identified 12 gene sets that remained significant after Bonferroni correction, led by inflammatory response, T-helper 17 (Th17) cell differentiation, regulation of inflammatory response, and inflammatory bowel disease signaling (Supplementary file Table S4). Gene set analysis further suggested that these genes play an important role in the immune system and inflammatory responses. Gene-property analysis further identified significant cross-trait enrichment in whole blood and small intestine after Bonferroni correction (Figures 2a and 2b; and Supplementary file Table S5).

### Pleiotropic loci shared by SD and CD

**Figure 3:**
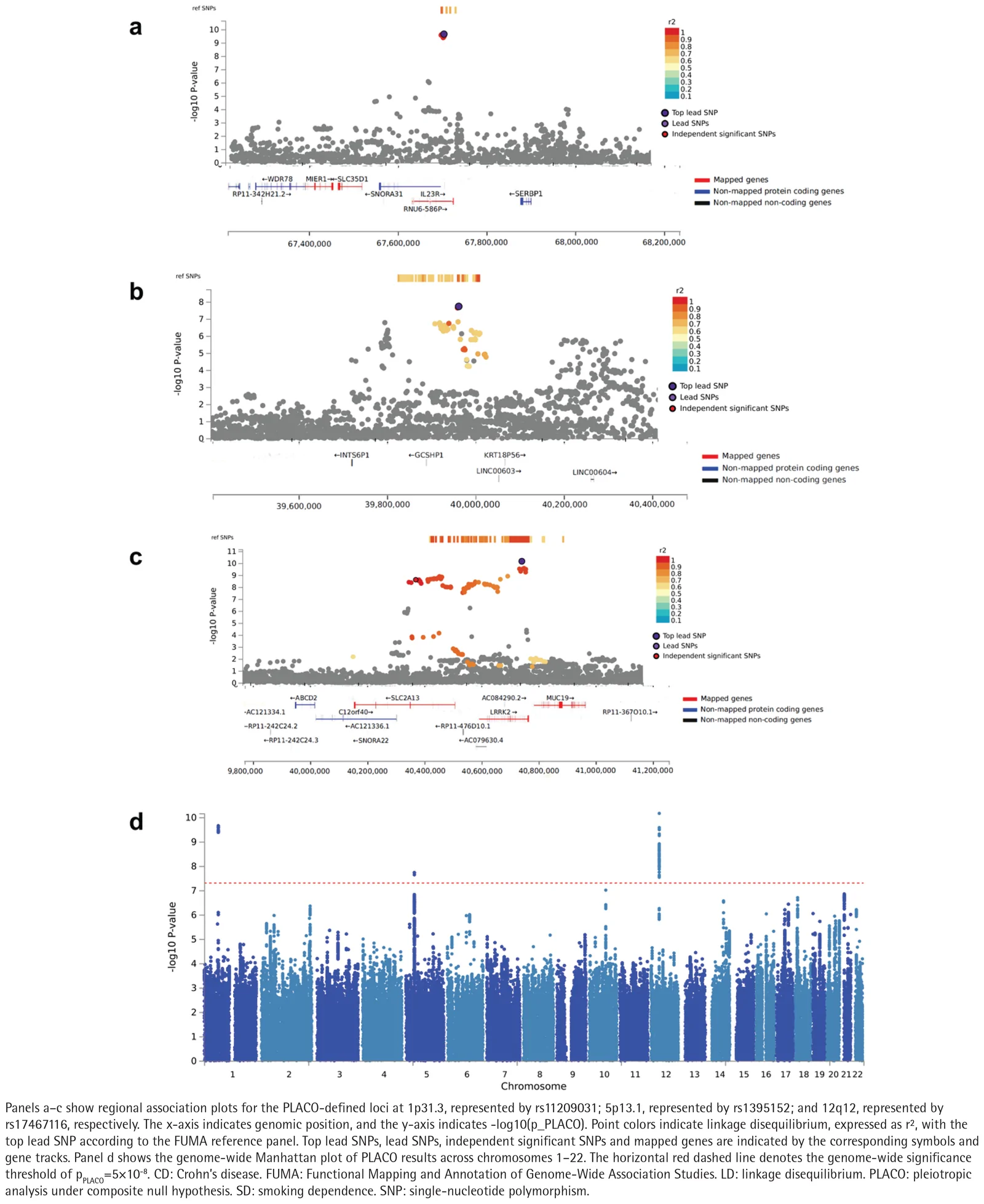
Shared pleiotropic loci between Crohn’s disease and smoking dependence in a cross-trait GWAS summary-statistics analysis of European-ancestry datasets (CD: GWAS Catalog GCST004132, 2017, N=40266; SD: FinnGen R11, 2024, N=452209)

In light of the significant genetic correlation between SD and CD detected by LDSC and HDL, we used pleiotropic analysis under the composite null hypothesis (PLACO) to identify loci that may influence both conditions. PLACO identified 81 SNPs associated with SD and CD at genome-wide significance (p_PLACO_ <5×10^-8^). FUMA consolidated these variants into three pleiotropic loci at 1p31.3, 5p13.1, and 12q12, represented by rs11209031, rs1395152, and rs17467116, respectively (Figure 3, Table 2). Although the PP4/PP3 ratios for rs11209031 and rs17467116 exceeded 2.5, the absolute posterior support for H4 remained insufficient, and none met the prespecified criteria for moderate or strong colocalization. The observed overlap therefore remained suggestive but inconclusive and may reflect partially shared or distinct causal effects rather than a single fully shared causal variant (Supplementary file Table S6). FUMA mapping annotated candidate genes including *MUC19*, *LRRK2*, *SLC2A13*, *MIER1*, *SLC35D1*, *IL23R*, *TTC33*, *PTGER4*, and *DAB2* (Supplementary file Table S7).

**Table 2:** Shared pleiotropic loci between Crohn’s disease and smoking dependence identified by PLACO and annotated using FUMA in a cross-trait analysis of European-ancestry GWAS summary statistics (CD: GWAS Catalog GCST004132, 2017, N=40266; SD: FinnGen R11, 2024, N=452209)

*CHR*	*rsID*	*pos*	*p*	*start*	*end*	*nSNPs*	*LeadSNPs*
1	rs11209031	67739588	2.223E-10	67731614	67764815	13	rs11209031
5	rs1395152	40000779	1.859E-08	39854168	40066269	130	rs1395152
12	rs17467116	40806737	6.867E-11	40180900	40956365	418	rs17467116

Genome-wide significant pleiotropic variants were identified using PLACO (p<5×10^-8^), and genomic risk loci were subsequently defined and annotated using FUMA. CD: Crohn’s disease. SD: smoking dependence. GWAS: genome-wide association study. PLACO: pleiotropic analysis under the composite null hypothesis. FUMA: Functional Mapping and Annotation. CHR: chromosome. rsID: reference SNP identifier. Pos: genomic position. p: PLACO p-value; start and end, locus boundaries. nSNPs: number of SNPs. LeadSNPs: lead variants.

### Integrated prioritization of candidate genes

The 22 genes that reached FDR significance in the MAGMA analysis were carried forward for SMR/HEIDI evaluation. Of these, 21 were testable in at least one prespecified core eQTL panel, whereas *RP4-583P15.14* (ENSG00000273047) had no matching probe in the core panels and was therefore not evaluated in the primary SMR/HEIDI analysis (Supplementary file Table S8). *RPS6KB1* showed corrected same-tissue expression-linked associations with both SD and CD in the PsychENCODE prefrontal cortex PEER50 panel, using the same probe and top cis-eQTL for both traits. The associations were directionally concordant (SD: β= -0.626, PSMR=0.0187, adjusted p=0.0411; CD: β= -0.358, PSMR=0.0217, adjusted p=0.0467), with no evidence of HEIDI heterogeneity (p_HEIDI=0.592 and 0.107, respectively). At a secondary evidence level, *MARK3* in transverse colon and *ARFRP1* in BrainMeta cortex showed nominal, directionally concordant same-tissue associations for both traits, but the SD associations did not remain significant after multiple-testing correction. *ZGPAT* also showed nominal same-tissue evidence in BrainMeta cortex. Complete results are provided in Supplementary file Table S8.

### Spatial mapping of cross-trait genetic signals and prioritized genes

**Figure 4:**
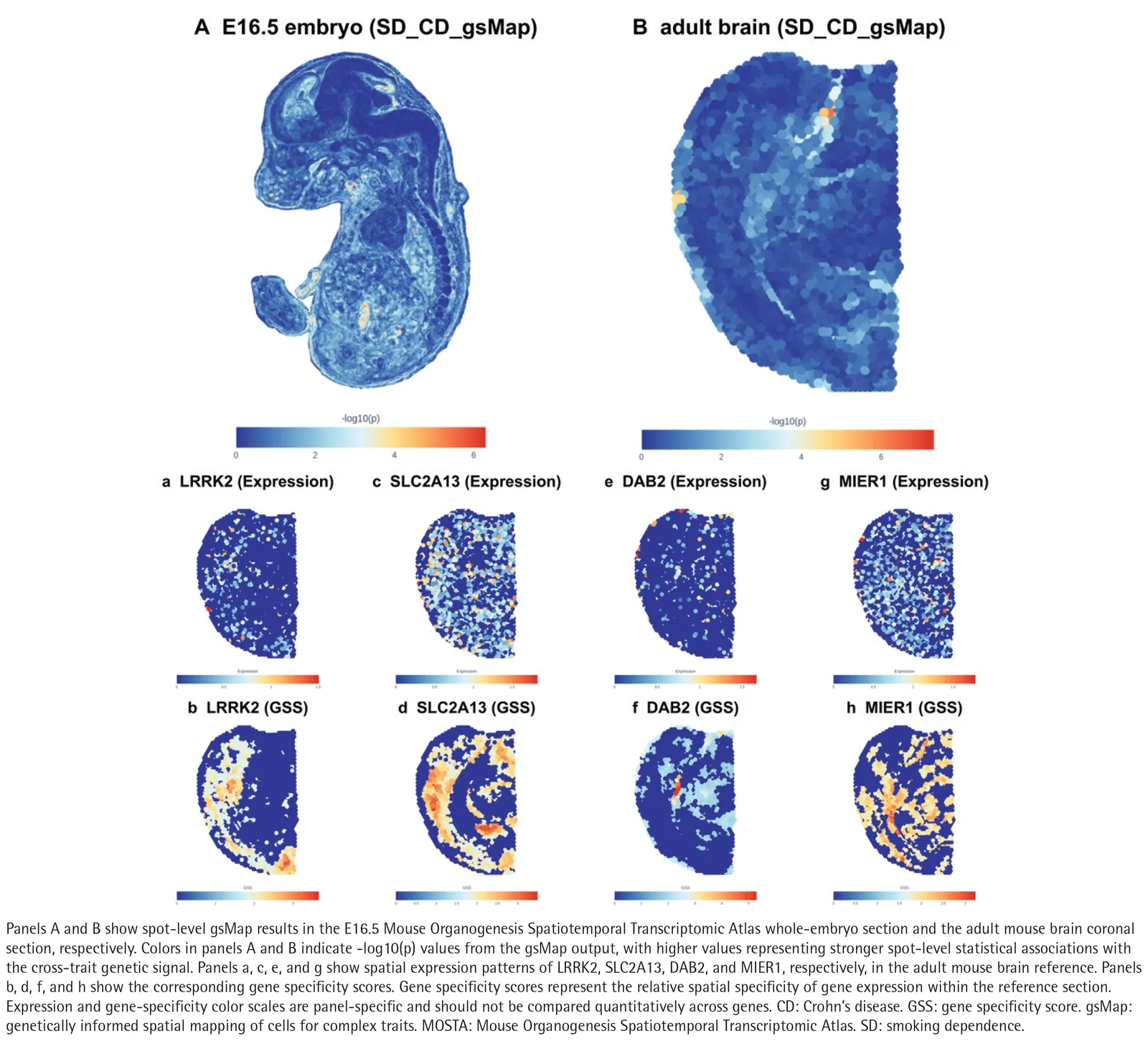
Spatial mapping of cross-trait genetic signals and prioritized genes for Crohn’s disease and smoking dependence in the analysis of European-ancestry GWAS summary statistics (CD: GWAS Catalog GCST004132, 2017, N=40266; SD: FinnGen R11, 2024, N=452209)

Spatial transcriptomic profiling of cross-trait genetic signals revealed distinct spatial association patterns across embryonic and adult mouse tissues (Figures 4A and 4B). Gene-level diagnostic results for the E16.5 whole-embryo reference are provided in Supplementary file Table S9, while the cross-trait signal showed a prominent spatial association in the developing intestinal tract in the corresponding spot-level analysis (Supplementary file Table S10). In the adult mouse brain reference, gene-level diagnostic results are provided in Supplementary file Table S11, and spot-level analysis revealed heterogeneous regional association patterns (Supplementary file Table S12), highlighting distinct intestinal and neural spatial contexts for the shared genetic architecture of CD and SD. Gene-level maps of the four selected FUMA-mapped genes – *LRRK2, SLC2A13, DAB2,* and *MIER1* – showed distinct spatial expression and GSS patterns in the adult mouse brain reference (Figures 4a–4h; and Supplementary file Table S11). These spatial findings provide additional context for the genetic signals shared between SD and CD.

## DISCUSSION

In this study, we applied an integrative cross-trait GWAS summary-statistics framework to characterize the shared genetic signal between SD and CD. LDSC and HDL identified a significant positive genome-wide genetic correlation, while PLACO identified 81 genome-wide significant pleiotropic SNPs, which FUMA mapped to three genomic loci. However, none of these loci met the prespecified criteria for moderate or strong colocalization. Functional analyses further implicated immune-inflammatory processes, particularly Th17-cell differentiation, with corresponding signals in blood and intestinal tissues. SMR/HEIDI analysis prioritized *RPS6KB1* as the strongest shared expression-linked candidate, whereas *LRRK2* remained the most prominent candidate supported by locus-level, MAGMA, and spatial evidence. Exploratory gsMap analysis further showed a prominent spatial association in the embryonic gastrointestinal tract and heterogeneous spatial association patterns in the adult mouse brain. Collectively, these findings support measurable shared genetic susceptibility between SD and CD but do not establish causal direction.

At the genome-wide level, the concordant LDSC and HDL results provide consistent evidence of shared polygenic susceptibility between SD and CD. These results are compatible with epidemiological evidence linking cigarette-smoking exposure with the development and clinical course of CD^27^. Previous studies have shown that smoking cessation is one of the primary non-pharmacological interventions for patients with CD, and that post-operative cessation substantially reduces the long-term risk of recurrence after ileocolic resection^28^. Taken together, these lines of evidence indicate that, beyond the therapeutic effect of smoking cessation itself, SD – as a behavioral phenotype partly influenced by genetic factors – may also be related to individual susceptibility to CD through shared genetic factors.

Although colocalization support across the three pleiotropic loci was limited, the 12q12 locus harboring *LRRK2* remained of particular interest. Previous genetic studies have established *LRRK2* as a susceptibility gene for CD. Mechanistically, *LRRK2* has been implicated in intestinal immune regulation: *LRRK2* deficiency enhances susceptibility to experimental colitis through dysregulation of NFAT signaling^29^, while more recent evidence indicates that hyperactive CD-associated *LRRK2* variants can impair macrophage autophagy and promote inflammatory signaling associated with Paneth-cell dysfunction^30^. Beyond the intestine, the *LRRK2* G2019S variant has been shown to alter vesicle trafficking, dopamine-receptor localization, and extracellular dopamine responses following nicotine stimulation in neuronal models^31^. Although these studies do not establish a direct role of *LRRK2* in smoking dependence, evidence from intestinal immune and dopaminergic neuronal systems provides biological context for its recurrent appearance in our cross-trait analyses. In our study, *LRRK2* was supported across the 12q12 pleiotropic locus, MAGMA gene-level association, and spatial mapping. This convergence across complementary analytical levels prioritizes *LRRK2* for further investigation; however, its SMR estimates in the whole-blood panel shown in Table 3 were opposite in direction for SD and CD, and same-tissue shared SMR/HEIDI support was not established, so these findings do not establish its causal involvement in either phenotype.

**Table 3:** Integrated gene-level and expression-linked evidence for prioritized genes in Crohn’s disease and smoking dependence: a cross-trait analysis of European-ancestry GWAS summary statistics (CD: GWAS Catalog GCST004132, 2017, N=40266; SD: FinnGen R11, 2024, N=452209)

*Gene*	*MAGMA evidence*	*eQTL panel*	*ProbeID/top SNP*	*SD β_SMR (SE)*	*SD p_SMR/FDR*	*SD p_HEIDI*	*CD β_SMR (SE)*	*CD p_SMR/FDR*	*CD p_HEIDI*
**RPS6KB1**	p=8.46×10^-6^ FDR=0.0175 p_bon_=0.1548	PsychENCODE prefrontal cortex PEER50	ENSG00000108443/rs4047777	-0.626 (0.266)	0.0187/0.0411	0.5919	-0.358 (0.156)	0.0217/0.0467	0.1075
**MARK3**	p=1.07×10^-5^ FDR=0.0178 p_bon_=0.1958	GTEx V8 Colon Transverse	ENSG00000075413/rs28444993	−0.205 (0.097)	0.0351/0.0741	0.2548	-0.260 (0.062)	2.48×10^-5^/1.66×10^-4^	0.2239
**ARFRP1**	p=3.69×10^-6^ FDR=0.0121 p_bon_=0.0676	BrainMeta v2 cortex cis-eQTL	ENSG00000101246.20/rs6062498	0.126 (0.064)	0.0477/0.0937	0.6645	0.254 (0.040)	2.03×10^-10^/4.62×10^-9^	0.1493
**LRRK2**	p=8.37×10^-7^ FDR=0.0100 p_bon_=0.0153	GTEx V8 Whole Blood	ENSG00000188906/rs1472118	-0.067 (0.242)	0.7815/0.8734	0.0785	0.458 (0.150)	0.0023/0.0079	3.11×10^-4^
**TNFRSF6B**	p=1.12×10^-6^ FDR=0.0100 p_bon_=0.0205	BrainMeta v2 cortex cis-eQTL	ENSG00000243509.6/rs2738783	-0.194 (0.174)	0.2647/0.3869	0.3485	-0.569 (0.141)	5.29×10^-5^/2.90×10^-4^	0.0485
**ZGPAT**	p=2.09×10^-6^ FDR=0.0100 p_bon_=0.0383	BrainMeta v2 cortex cis-eQTL	ENSG00000197114.12/rs1291213	0.241 (0.122)	0.0473/0.0937	0.2683	0.404 (0.085)	1.83×10^-6^/1.74×10^-5^	0.1781
**RP4-583P15.15**	p=2.19×10^-6^ FDR= 0.0100 p_bon_=0.0401	PsychENCODE prefrontal cortex PEER50	ENSG00000273154/rs2872881	0.320 (0.155)	0.0393/0.0799	0.3946	0.525 (0.096)	4.44×10^-8^/7.22×10^-7^	0.0050

SMR and HEIDI analyses were used to evaluate expression-linked support for prioritized genes across available eQTL datasets. FDR-adjusted p-values were calculated using the Benjamini–Hochberg procedure. HEIDI testing was used to assess heterogeneity, with p_HEIDI >0.05 indicating no statistically significant heterogeneity. SMR/HEIDI findings were interpreted as expression-linked evidence rather than evidence of causality. RPS6KB1 showed corrected same-tissue shared SMR/HEIDI support for both traits using the same PsychENCODE prefrontal cortex PEER50 panel, probe, and top SNP. MARK3 in transverse colon and ARFRP1 in BrainMeta cortex showed directionally concordant same-tissue associations for both traits at the nominal level, but their SD associations did not remain significant after pooled BH correction; they are therefore reported as secondary rather than corrected shared evidence. ZGPAT showed nominal same-tissue evidence that was not robust across HEIDI sensitivity analyses, whereas LRRK2, TNFRSF6B, and RP4-583P15.15 did not show convergent shared SMR/HEIDI support. SD: smoking dependence. CD: Crohn’s disease. MAGMA: Multi-marker Analysis of GenoMic Annotation. SMR: summary-data-based Mendelian randomization. HEIDI: heterogeneity in dependent instruments. FDR: false discovery rate. eQTL: expression quantitative trait locus. SE: standard error. SNP: single-nucleotide polymorphism.

Tissue and pathway analyses converged on an immune–intestinal pattern. In our study, the convergence of blood and intestinal tissue signals with prominent inflammatory-response and Th17-cell differentiation pathways suggests that part of the genetic overlap between SD and CD may involve regulatory programs operating at the interface between systemic immunity and intestinal mucosal inflammation. This pattern is consistent with recent single-cell genetic studies showing that IBD risk variants exert cell-type-dependent regulatory effects in blood and intestinal tissues^32^. Against this background, the Th17 signal provides a more specific immunological axis through which the tissue-level findings may be interpreted. Previous experimental evidence indicates that cigarette-smoke exposure can enhance Th17 responses and aggravate intestinal inflammation^33^, while mucosal studies in CD have independently implicated the IL-23/Th17 axis in intestinal inflammatory responses^34^. Taken together, the convergence of blood- and intestinal-tissue associations with inflammatory and Th17-related gene sets provides a coherent immune–intestinal context for the shared genetic signal. However, these enrichment analyses do not establish that these tissues or pathways mediate the genetic relationship between SD and CD.

SMR/HEIDI analysis further prioritized *RPS6KB1* as the strongest shared expression-linked candidate between SD and CD, with directionally concordant associations in the same prefrontal-cortex eQTL panel that remained significant after multiple-testing correction and showed no evidence of HEIDI heterogeneity. *RPS6KB1* encodes S6 kinase 1 (S6K1), a major downstream effector of mTORC1. Experimental studies have shown that the PI3K–Akt–mTORC1–S6K1/2 axis regulates Th17 differentiation^35^, providing a biological link to the Th17-related signals identified in our functional analyses. Beyond immune regulation, S6K1 signaling in the prefrontal cortex has been experimentally linked to synaptic protein synthesis and neuronal responses^36^, which is notable given that our shared SMR signal was detected in a prefrontal-cortex eQTL panel. Although direct evidence linking *RPS6KB1* to nicotine dependence remains limited, these observations provide biological context for the shared expression-linked signal identified in our analysis. The SMR result should be interpreted as expression-linked prioritization rather than proof that altered *RPS6KB1* expression causally affects either phenotype.

Finally, exploratory gsMap analysis revealed a prominent spatial association of the shared SD–CD genetic signal in the gastrointestinal tract of the E16.5 mouse embryo, together with heterogeneous spatial association patterns in the adult mouse brain. The presence of signals in both intestinal and neural spatial contexts is notable given the inflammatory nature of CD and the neurobehavioral basis of SD, and suggests that their shared genetic susceptibility may involve distinct cellular environments across the gut and brain. However, these maps should be interpreted as spatial contextualization. In particular, the use of mouse developmental and adult spatial references limits direct inference regarding disease-relevant cellular states in human intestinal or neural tissues.

### Limitations

Several limitations should be acknowledged. First, the GWAS data used in this study were restricted to individuals of European ancestry, and replication in independent multi-ethnic cohorts is required before broader generalization. Second, smoking dependence in FinnGen was defined using registry-based diagnostic codes, which may introduce phenotype heterogeneity and misclassification. Third, our analyses of genetic correlation and pleiotropy were based on summary statistics and therefore cannot establish causality. Fourth, we did not explicitly model gene–environment interactions, and thus could not quantify the strength of interaction between smoking exposure and genetic variation. SMR/HEIDI cannot prove expression mediation, and gsMap used mouse spatial references without functional validation. Finally, the specific biological mechanisms linking SD and CD remain to be clarified. Future work using cellular and animal models will be necessary to test the associations identified here.

## CONCLUSIONS

Our findings provide evidence that SD and CD share an underlying genetic susceptibility. This suggests that their observed association may partly reflect common inherited factors, alongside the established effects of smoking exposure. The shared genetic architecture also points to biological connections between smoking dependence and intestinal inflammation that remain to be defined. These findings broaden the genetic perspective on the relationship between smoking and CD and provide a basis for defining the biological processes underlying this association.

## Supplementary Material



## Data Availability

The GWAS summary statistics supporting this study are publicly available from the NHGRI-EBI GWAS Catalog (GCST004132; https://www.ebi.ac.uk/gwas/studies/GCST004132)^8^ and FinnGen release R11 (SMOKING_DEPEND; https://r11.finngen.fi/pheno/SMOKING_DEPEND)^9^.
